# Additive effect of aortic regurgitation degree on left ventricular strain in patients with type 2 diabetes mellitus evaluated via cardiac magnetic resonance tissue tracking

**DOI:** 10.1186/s12933-022-01471-2

**Published:** 2022-03-11

**Authors:** Li-Ting Shen, Li Jiang, Ya-Wen Zhu, Meng-Ting Shen, Shan Huang, Rui Shi, Yuan Li, Zhi-Gang Yang

**Affiliations:** 1 Department of Radiology, West China Hospital, Sichuan University, 37# Guo Xue Xiang, Chengdu, 610041 Sichuan China; 2 Division of General Practice, West China Hospital, Sichuan University, Chengdu, Sichuan China

**Keywords:** Type 2 diabetes mellitus, Aortic regurgitation, Left ventricle, Strain, Magnetic resonance imaging

## Abstract

**Background:**

Type 2 diabetes mellitus causes left ventricular (LV) remodeling and increases the risk of aortic regurgitation (AR), which causes further heart damage. This study aimed to investigate whether AR aggravates LV deformation dysfunction and to identify independent factors affecting the global peak strain (PS) of LV remodeling in patients with type 2 diabetes mellitus (T2DM) who presented with AR and those without T2DM.

**Methods:**

In total, 215 patients with T2DM and 83 age- and sex-matched healthy controls who underwent cardiac magnetic resonance examination were included. Based on the echocardiogram findings, T2DM patients with AR were divided into three groups (mild AR [n = 28], moderate AR [n = 21], and severe AR [n = 17]). LV function and global strain parameters were compared, and multivariate analysis was performed to identify the independent indicators of LV PS.

**Results:**

The T2DM patients with AR had a lower LV global PS, peak systolic strain rate (PSSR), and peak diastolic strain rate (PDSR) in three directions than those without AR and non-T2DM controls. Patients without AR had a lower PS (radial and longitudinal) and PDSR in three directions and higher PSSR (radial and longitudinal) than healthy controls. Further, regurgitation degree was an independent factor of LV global radial, circumferential, and longitudinal PS.

**Conclusion:**

AR may aggravate LV stiffness in patients with T2DM, resulting in lower LV strain and function. Regurgitation degree and sex were independently correlated with LV global PS in patients with T2DM and AR.

**Supplementary Information:**

The online version contains supplementary material available at 10.1186/s12933-022-01471-2.

## Background

Type 2 diabetes mellitus (T2DM) and its related cardiovascular diseases are causing increasing burden in terms of morbidity and mortality. T2DM is characterized by insufficient or non-effective utilization of insulin, resulting in chronic hyperglycemia [1, 2]. Aortic regurgitation (AR) can occur if diabetes causes left ventricular (LV) remodeling and increases the risk of degenerative aortic valve disease. This phenomenon induces LV load and a series of heart damage [3]. Except for the frequently studied risk factors, T2DM, which is associated with a higher risk of AR, is an incremental challenge in the future, even if most patients are asymptomatic and often neglected. Changes in cardiac structure, function, and strain in patients with T2DM who present with AR are not completely understood and are challenging to quantify. Previous studies have investigated the independent effects of T2DM or AR as separate entities on cardiac structure and function [4, 5]. In addition, the comprehensive effects based on cardiac strain have not been fully evaluated. Therefore, it is important to investigate cardiac dysfunction in patients with T2DM who present with AR before the occurrence of adverse events to reduce cardiovascular risk and improve outcomes.

Cardiac magnetic resonance (CMR) tissue-tracking technology, which is based on the post-processing of standard steady-state free precession cine images, has become a well-accepted imaging tool for quantifying diabetes-related cardiac deformation dysfunction [6–8]. CMR can provide unique information about LV strain in subclinical conditions and recognize structural and functional changes sensitively before LV ejection fraction (LVEF) or dimension changes [9]. Moreover, it can identify early signals for the diagnosis and treatment of myocardial injury quantitatively and digitally [10, 11]. Thus, this study aimed to compare the characteristics of LV structure, function, and strain, to investigate whether AR aggravates LV deformation dysfunction, and to identify independent factors affecting the global peak strain (PS) of LV in patients with T2DM who present with AR and those without T2DM.

## Methods

### Study population

This study was approved by the Biomedical Research Ethics Committee of our hospital and conducted in accordance with the Declaration of Helsinki (2013 Edition). Due to the retrospective nature of the research, the need for informed consent was waived.

This research included 432 individuals with T2DM who were clinically diagnosed based on the current American Diabetes Association guidelines and who underwent CMR examinations at our hospital between April 2012 and April 2021 [12]. The exclusion criteria were patients with a history of primary cardiomyopathy, congenital heart disease, myocardial infarction, rheumatic heart disease, aortic valve stenosis, aortic valve stenosis accompanied regurgitation, aortic valve surgery history, other severe non-AR valve regurgitation diseases and those with a poor image quality for post-processing analysis. Finally, 215 patients who met these criteria were included in the analysis and were categorized into two groups according to the presence or absence of AR: those with T2DM but without AR (T2DM [AR −]) (n = 149) and those with T2DM who presented with AR (T2DM [AR +]) (n = 66). The diagnosis of AR and regurgitant degree were based on echocardiography (Additional file 2: Fig. S2). The average age and body mass index (BMI) of patients with diabetes were 54 (range: 48–60) years and 22.96 (range: 21.03–26.00) kg/m^2^, respectively. Among them, 66 (37.6%) presented with AR (n = 28, mild; n = 21, moderate; and n = 17, severe), whereas 149 (62.4%) did not. Concurrently, 83 individuals with sex, age, and BMI distribution similar to those of our patients were included in the control group. All patients and controls underwent the same CMR examination.

### Basic information and laboratory data collection

Data regarding age, sex, disease duration, blood pressure, height, and weight were extracted, and BMI was calculated. In addition, we collected the following laboratory and clinical data: fasting blood glucose, glycated hemoglobin, triglyceride, total cholesterol, high-density lipoprotein and low-density lipoprotein cholesterol levels, duration of diabetes (years), and use of antidiabetic drugs (α-glucosidase inhibitors, biguanides, sulfonylureas, glucagon-like peptide-1/dipeptidyl peptidase-4 inhibitors, sodium-glucose cotransporter 2 inhibitors, and insulin). The baseline information of healthy controls was collected before CMR scanning.

### CMR protocol

A 3.0-T whole-body scanner (Skyra; Siemens Medical Solutions, Erlangen, Germany) with a 32-channel body phased-array coil was used to examine patients while in the supine position. Data were acquired during the breath-holding period at the end of inspiration. A series of 8–12 consequent short-axis views of the LV from the aortic valve to the level of the LV apex were obtained using steady-state free precession with the following parameters: temporal resolution, 39.34 ms; echo time, 1.22 ms; slice thickness, 8.0 mm; flip angle, 39°; field of view, 234 × 280 mm^2^; 1.31 ms; and matrix size, 208 × 139 pixels.

### Image analysis

#### Calculation of LV volume and functional parameters

LV volume was calculated, and functional parameter image analysis was performed using commercial software (cvi42; Circle Cardiovascular Imaging, Inc., Calgary, AB, Canada) by two skilled radiologists with > 3 years of CMR experience. The endo- and epi-cardial contours of the LV were contoured manually per slice on the end-diastole and end-systole images, and the papillary muscles and moderator bands were cautiously excluded. The LV volume and functional parameters, including end-diastolic volume, end-systolic volume (ESV), LV stroke volume (LVSV), LVEF, and LV mass were automatically calculated (Additional file 1: Fig. S1).

#### Identification and classification of AR in patients with T2DM

Echocardiography could facilitate the quantitative calculation of regurgitant volume and fraction, which can equally provide a better quantization of AR severity [13–15]. Color flow Doppler ultrasonography of the parasternal long-axis view provides an estimate of the regurgitant orifice size. The color jet diameter (or width) immediately below (within 1 cm) the aortic valve was measured in diastole. The jet diameter-to-left ventricular outflow tract (LVOT) diameter ratio was calculated as Jet/LVOT width (%). This ratio helps assess the degree of regurgitation: trace, < 10%; mild, 10–24%; moderate, 25–65%; and severe, > 65% [16, 17] (Additional file 2: Fig. S2).

### LV strain analysis

Each voxel of the myocardium was pursued on the horizontal four-chamber long-axis, vertical two-chamber long-axis, and short-axis cine slices. The software automatically analyzed the global LV strain variables, including PS, peak systolic strain rate (PSSR), and peak diastolic strain rate (PDSR). Each strain parameter had three values in different directions (radial, circumferential, and longitudinal). The radial strain had a positive value due to myocardium thickening during LV contraction. The circumferential and longitudinal strains had negative values because the myocardium shortened during contraction [18, 19].

### Reproducibility of LV strain

The intraobserver variability of LV global strain parameters was obtained by an experienced investigator by comparing the measurements in 80 randomly selected cases analyzed by the same observer after 1 month. The interobserver variability was evaluated by comparing the measurements from the same group by another independent double-blinded skilled observer.

### Statistical analysis

Continuous data were assessed using the Shapiro–Wilk test and the Levene variance test to assess for distribution. Continuous variables, including low-density lipoprotein cholesterol level, eGFR, LVEF, and circumferential and radial PS were expressed as mean ± standard deviation. Moreover, the longitudinal PS among the three groups was compared via a single factor analysis of variance. The median (interquartile range: 25–75%) was calculated for those with non-normal distribution. The Kruskal–Wallis H test was used for a multi-component comparison, and the Mann–Whitney U test was utilized to compare patients with T2DM who presented with AR and those without. Spearman’s correlation was applied to analyze the correlation between LV global PS (radial, circumferential, and longitudinal), regurgitation degree, duration of diabetes, and blood lipid levels. The absolute values of strains (circumferential and longitudinal) were used in the correlation analysis to eliminate confusion caused by the negative value. Variables with a P value of < 0.1 and the absence of collinearity in the univariate analysis were included in the stepwise multiple linear regression model adjusted for sex, age, BMI, systolic blood pressure, and resting heart rate. Inter- and intra-observer variabilities were assessed using intraclass correlation coefficient (ICC). All analyses were performed using a two-tailed test, and a P value of < 0.05 was considered statistically significant. All analyses were performed using the Statistical Package for the Social Sciences software (version 24.0; IBM, Armonk, New York, the USA) and GraphPad Prism (version 7.0a; GraphPad Software, San Diego, California, the USA).

## Results

### Study population and baseline characteristics of participants

Overall, 298 individuals were included in the study. Among them, 149 were T2DM (AR −); 66 were T2DM(AR +); and 83 were healthy controls. The mean age of the participants was 58 (range: 52–66) years, and 78 (31.5%) were women. Table 1 shows the general characteristics of the participants. All patients with T2DM were older and had a higher systolic blood pressure than the healthy controls (all P < 0.05). Compared with the T2DM(AR −) group, the T2DM(AR +) group was older (68 [range: 61–75] vs. 58 [range: 52–65] years) (P < 0.001) and had had lower glycosylated hemoglobin values (6.70% [range: 6.40–7.15%] vs. 7.20% [range: 6.60–8.28%]) (P = 0.001) and lower fasting plasma glucose levels (6.97 [range: 5.87–9.10] vs. 8.59 [range: 6.42–10.65] mmol/L).

**Table 1:** Demographic and clinical variables of study cohort

	Controls (n = 83)	T2DM (AR −) (n = 149)	T2DM (AR +) (n = 66)
Age (years)	62 (55,69)	58 (52, 65)*	68 (61, 75)*†
Gender(% female)	28 (33.73)	49 (32.88)	24 (36.36)
BMI(kg/m^2^)	24.2 (22.6, 26.5)	24.3 (22.5, 26.4)*	24.0 (22.9, 26.3)
Restheart rate (bpm)	76 (67, 86)	75 (65, 84)	77 (70, 88)
SBP (mmHg)	120.0 (116.0, 124.0)	128.0 (116.0, 140.0)*	128.0 (118.0, 140.5)*
DBP (mmHg)	79.0 (70.0, 87.0)	78.00 (70.0, 87.0)	80.0 (70.0, 87.0)
Fasting plasma glucose, mmol/l	–	8.59 (6.42, 10.65)	6.97 (5.87, 9.10)†
HbA1c (%)	–	7.20 (6.60, 8.28)	6.70 (6.40, 7.15)†
Diabetes duration (year)	–	7.25 (4.00, 12.00)	8.50 (3.50, 12.00)
TG (mmol/l)	–	1.37 (1.05, 2.00)	1.29 (1.10, 1.60)
TC (mmol/l)	–	4.24 (3.54, 5.07)	3.72 (3.14, 4.79)
HDL (mmol/l)	–	1.09 (0.87, 1.35)	1.21 (1.02, 1.48)
LDL (mmol/l)	–	2.42 ± 0.96	2.23 ± 0.81
Regurgitation degree			
Mild, n (%)	–	–	29 (43.9)
Moderate, n (%)	–	–	20 (30.3)
Severe, n (%)	–	–	17 (25.8)
Diabetes related drugs			
Biguanides, n (%)	–	37 (33.6)	10 (15.2)
Sulfonylureas, n (%)	–	21 (19.2)	19 (28.8)
α-Glucosides inhibitor, n (%)	–	24 (21.8)	20 (30.3)
GLP-1/DPP-4 inhibitor	–	7 (6.4)	5 (7.6)
Insulinum	–	14 (12.7)	12 (18.1)
Medication unknown	–	7 (6.3)	0
eGFR (mL/min)	–	72.3 ± 29.3	67.5 ± 19.7

*T2DM* type 2 diabetes mellitus, *AR* aortic regurgitation, *BMI* body mass index, *SBP* systolic blood pressure, *DBP* diastolic blood pressure, *HbA1c* glycated haemoglobin, *TC* total cholesterol, *TG* triglycerides, *HDL* high-density lipoprotein, *LDL* low-density lipoprotein, *eGFR* estimated glomerular filtration rate

*P < 0.05, T2DM vs. Normal; ^†^P < 0.05, T2DM with AR vs. T2DM without AR

### Characteristics of CMR-derived LV geometric, functional, and strain parameters in patients with T2DM who presented with AR and without AR

**Fig. 1:**
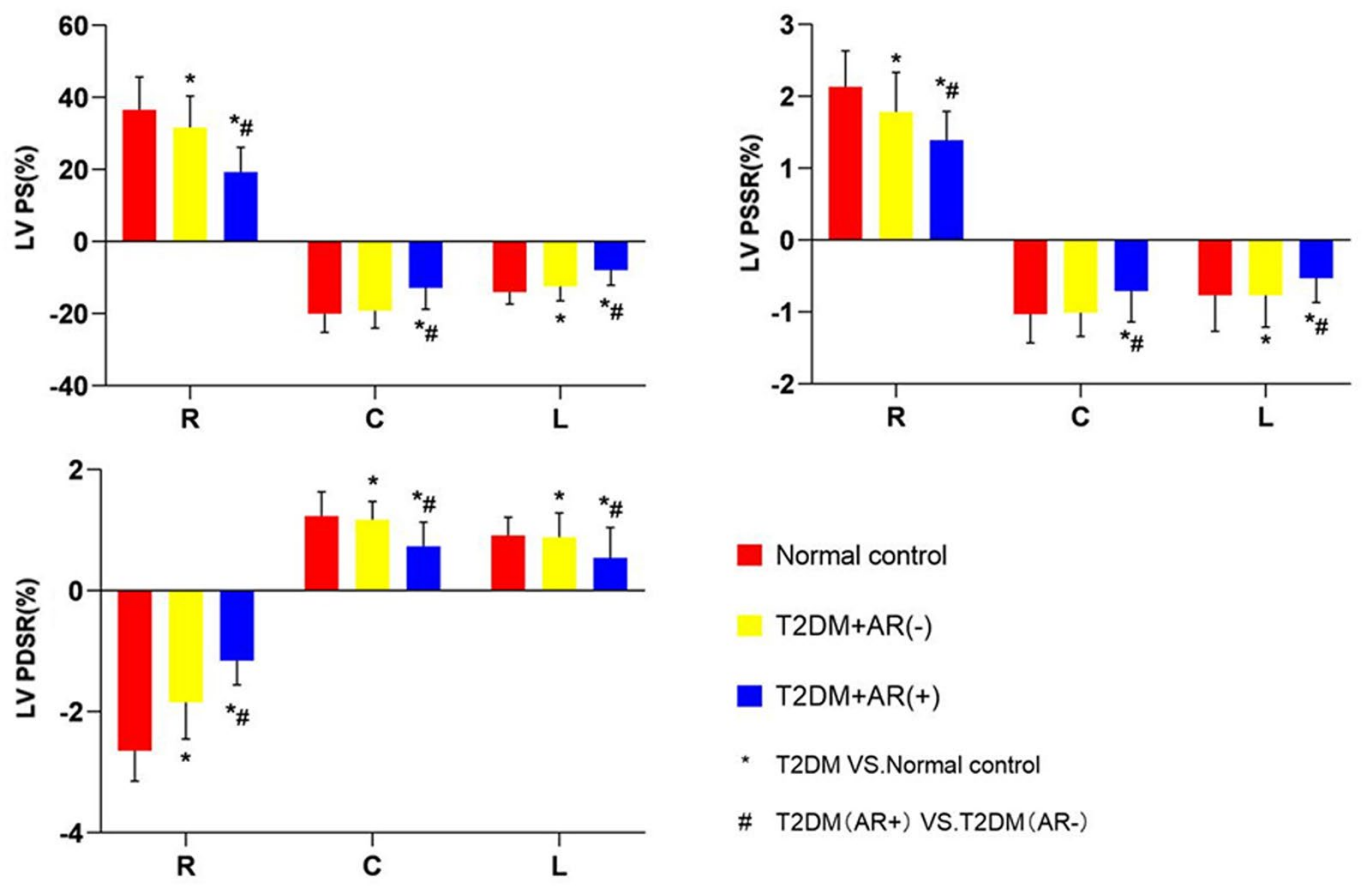
The CMR-derived LV strain parameters among controls, T2DM(AR-) and T2DM(AR +) in LV PS(%), PSSR(1/s), PDSR(1/s). *P < 0.05, T2DM vs. Normal; ^#^P < 0.05, T2DM patients with AR vs. T2DM patients without AR. T2DM, type2 diabetes mellitus; AR, aortic regurgitation; LV, left ventricular; PS, peak strain; PSSR, peak systolic strain rate; PDSR, peak diastolic strains rate

Tables 2 and 3 show the CMR findings, and Fig. 1 depicts the strain comparison of the three groups. Based on traditional LV function, the T2DM (AR +) group had lower LVEF and LVSV and higher LVESV, LVEDV, and LV mass index than the control and T2DM (AR −) groups (all P < 0.05). Compared with the control group, the LV mass index in T2DM (AR −) group was lower (AR [59.4 ± 12.1 g/m^2^] vs. [60.7 ± 11.4 g/m^2^]), but there existed no significant difference. Patients with T2DM who presented with AR had a lower LV global PS, PSSR, and PDSR in the three directions than patients without AR and healthy controls (all P < 0.05). The patients without AR had a lower PS (radial and longitudinal) and PDSR in the three directions and a higher PSSR (radial and longitudinal) than healthy controls (all P < 0.05).

**Table 2:** Comparison of CMR findings among T2DM patients with/without AR and normal controls

	Controls (n = 83)	T2DM (AR −) (n = 149)	T2DM (AR +) (n = 66)
LVEDV, mL	142.6 (115.6, 187.1)	135.5 (109.2, 161.0)	191.0 (136.2, 251.0)^*†^
LVESV, mL	58.7 (42.8, 95.1)	49.12 (38.0, 69.8)	102.6 (56.4, 195.6)^*†^
LVSV, mL	73.7 (59.9, 95.8)	78.3 (63.5, 96.6)	66.0 (48.2, 82.0)^*†^
LVEF, %	62.0 (41.5, 64.1)	61.4 (54.3, 66.7)	43.2 ± 2.6^*†^
LV mass index, (g/m^2^)	60.7 ± 11.4	59.4 ± 12.1	71.9 ± 29.3^*†^
PS (%)			
Radial	36.6 ± 9.5	29.9 (23.8, 37.4)^*^	15.3 (9.5, 27.6)^*†^
Circumferential	− 20.0 ± 3.0	− 19.0 ± 4.5	− 13.0 ± 5.9^*†^
Longitudinal	− 14.0 ± 3.4	− 12.2 ± 4.0^*^	− 7.9 ± 4.2^*†^
PSSR (1/s)			
Radial	1.54 (1.1, 2.0)	1.8 (1.3, 2.1)^*^	1.0 (0.7, 1.5)^*†^
Circumferential	− 0.9 (− 1.1, − 0.7)	− 1.0 ± 0.4	− 0.7 ± 0.3^*†^
Longitudinal	− 0.7 (− 0.8, − 0.5)	− 0.7 (− 0.9, − 0.5)^*^	− 0.5 (− 0.7, − 0.3)^*†^
PDSR (1/s)			
Radial	− 2.7 ± 0.9	− 1.8 (− 2.3, − 1.2)^*^	− 1.0 (− 1.6, − 0.6)^*†^
Circumferential	1.2 ± 0.3	1.1(0.9, 1.3)^*^	0.7 ± 0.3^*†^
Longitudinal	0.9 ± 0.3	0.7(0.5, 0.9)^*^	0.5(0.4, 0.6)^*†^

*P < 0.05, T2DM vs. Normal; ^†^P < 0.05, T2DM patients with AR vs. T2DM patients without AR. T2DM type 2 diabetes mellitus, *AR* aortic regurgitation, *LV* left ventricular, *EDV* end diastolic volume, *ESV* end systolic volume, *SV* stroke volume, *EF* ejection fraction, *PS* peak strain, *PSSR* peak systolic strain rate, *PDSR* peak diastolic strain rate

**Table 3:** Comparison of LV strain among T2DM patients with mild/moderate/severe regurgitation and normal controls

	Controls (n = 83)	Mild (n = 28)	Moderate(n = 21)	severe(n = 17)
PS (%)				
Radial	27.7 (17.6, 35.3)	27.3 (21.0, 34.2)^*^	14.1 (8.7, − 22.7)^*#^	6.8 (5.2, − 9.8)^*#^
Circumferential	− 18.2 (− 20.8, − 13.7)	− 17.0 ± 4.3^*^	− 12.2 ± 5.4^*#^	− 6.6 ± 2.9^*#^
Longitudinal	− 14.0 ± 3.37	− 11.5 ± 2.8^*^	− 6.8 ± 2.3^*#^	− 3.0 (− 4.4, − 2.0)^*#^
PSSR (1/s)				
Radial	1.5 (1.1, 2.0)	1.4 (1.2, 1.9)	0.9 (0.6, 1.4)^*#^	0.6 (0.4, 0.8)^*#^
Circumferential	− 0.9 (− 1.1, − 0.7)	− 0.84 (− 1.07, − 0.66)^*^	− 0.7 (− 0.8, − 0.5)^*#^	− 0.5 ± 0.2^*#^
Longitudinal	− 0.7 (− 0.8, − 0.5)	− 0.7 ± 0.4	− 0.5 (− 0.7, − 0.3)^*#^	− 0.3 (− 0.4, − 0.2)^*#^
PDSR (1/s)				
Radial	− 1.6 (− 2.1, − 1.0)	− 1.5 (− 2.1, − 1.0)^*^	− 1.0 (− 1.5, − 0.6)^*#^	− 0.5 (− 0.8, − 0.3)^*#^
Circumferential	1.2 ± 0.3	0.8 (0.6, 1.1)^*^	0.7 ± 0.3^*^	0.5 ± 0.2^*#^
Longitudinal	0.61 (0.45, 0.82)	0.62 (0.54, 0.76)^*^	0.45 (0.35, 0.51)^*#^	0.3 (0.2, 0.4)^*#^

*P < 0.05, T2DM with AR vs. normal; ^#^P < 0.05, T2DM with severe/moderate AR vs. T2DM with mild AR, *T2DM* type 2 diabetes mellitus, *AR* aortic regurgitation, *PS* peak strain, *PSSR* peak systolic strain rate, *PDSR* peak diastolic strain rate

### Characteristics of LV strain in the T2DM (AR +) group with different regurgitation degrees

**Fig. 2:**
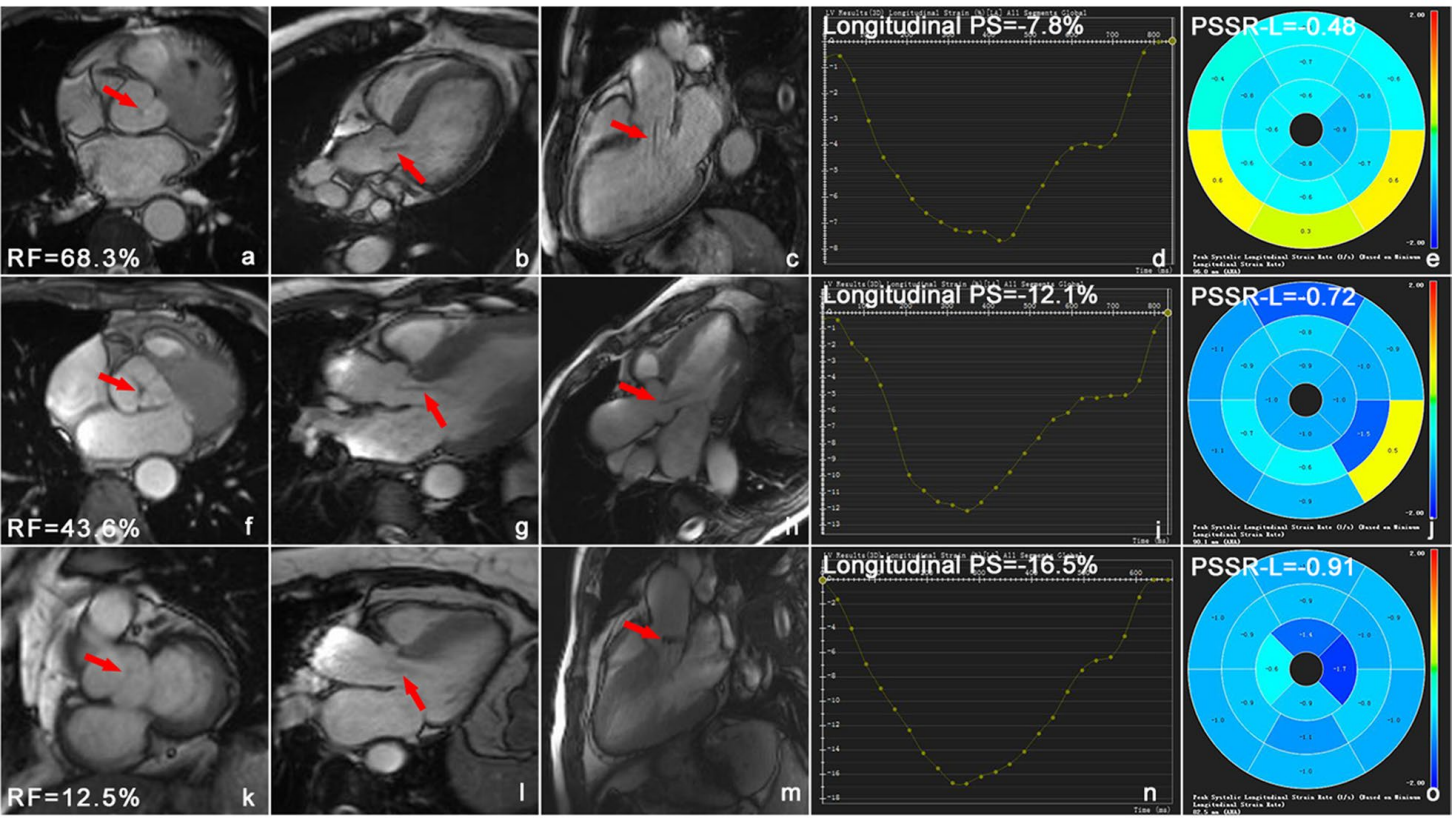
Cardiac cine images and three-dimensional pseudo-colour images of LV longitudinal strain in T2DM patients with mild, moderate and severe regurgitation. **a**–**c** T2DM patient with severe aortic regurgitation, male, 59 years old, left ventricular short axis (**a**), four-chamber (**b**), two-chamber (**c**), RF = 68.3%; **f**–**h**, T2DM with moderate aortic regurgitation, female, 52 years old, left ventricular short axis (**f**), four-chamber (**g**), two-chamber (**h**) cine sequence images showed moderate aortic regurgitation (red arrow), RF = 43.6%; k-m, T2DM patient with mild aortic regurgitation, male, 58 years old, left ventricular short axis (**k**), four-chamber (**l**), two-chamber (**m**), RF = 12.5%. **d**, **i**, **n** and **e**, **j**, **o** were three-dimensional pseudo color maps of left ventricular PS and PSSR in longitudinal. Cine sequence images showed black regurgitation signal from aorta into left ventricle (red arrow). *T2DM* type 2 diabetes mellitus, *RF* regurgitation fraction, *PS* peak strain, *PSSR* peak systolic strain rate

In the T2DM (AR +) group, 28 (43.9%) patients presented with mild, 21 (30.3%) with moderate, and 17 (25.8%) with severe AR. Table 3 shows the PS, PSSR, and PDSR of the three groups. Figure 2 shows the cardiac cine images and three-dimensional pseudo-color images of LV longitudinal strains of patients with T2DM who presented with mild, moderate, and severe regurgitation.

The mild AR group had lower PS (radial, circumferential, and longitudinal), PSSR (circumferential), and PDSR (radial, circumferential, and longitudinal) than the healthy control group (all P < 0.05). The moderate group had lower PS, PSSR, and PDSR in the three directions than the healthy control group (all P < 0.05). Compared with patients with mild AR, those with moderate and severe AR had a lower PS (radial, circumferential, and longitudinal), PDSR (radial and longitudinal), and PSSR (radial, circumferential, and longitudinal) (all P < 0.05). The severe AR group had significantly lower PS, PSSR, and PDSR in the three directions than the healthy control and mild AR groups (all P < 0.05).

### Factors influencing LV global PS in patients with T2DM who presented with AR

**Fig. 3:**
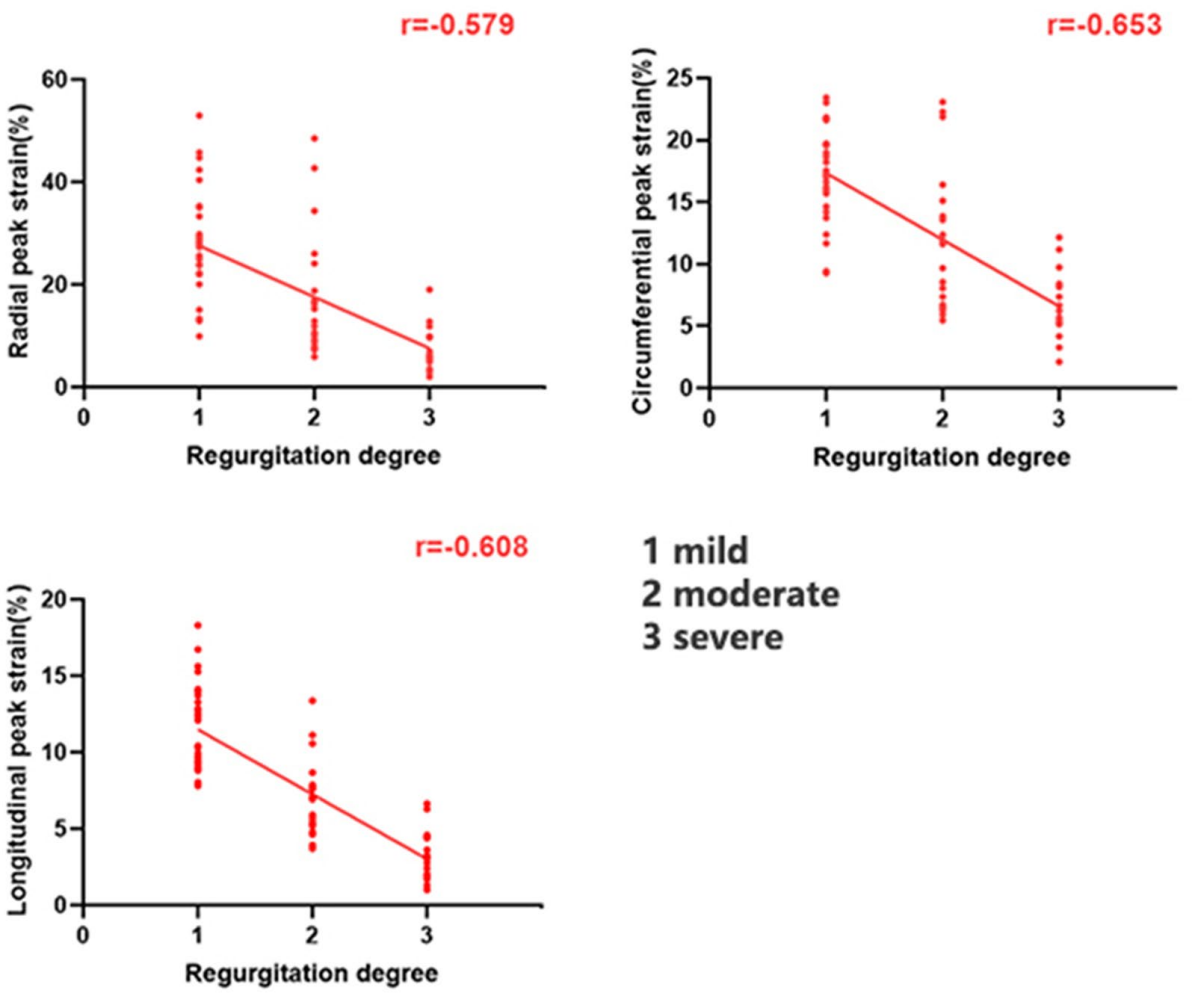
Correlation between the regurgitation degree of diabetes and LV global PS. The absolute value of PS was used in the circumferential and longitudinal direction analysis to avoid the influence of directional sign. *T2DM* type 2 diabetes mellitus, *r* correlation coefficient

There was a negative correlation between the AR degree and LV global radial PS (R = − 0.579, P < 0.001), circumferential PS (R = − 0.653, P < 0.001), and longitudinal PS (R = − 0.608, P < 0.001) in patients with T2DM who presented with AR. In addition, fasting plasma glucose level was negatively correlated with LV global radial PS (R = − 0.185, P = 0.071) and longitudinal PS (R = − 0.183, P = 0.019), but not circumferential PS (R = − 0.125, P = 0.111) in patients with T2DM who presented with AR (Table 4). Figure 3 shows the scatter plot illustrating the correlation between the regurgitation degree and LV global PS.

**Table 4:** Univariable and multivariable analysis between the magnitude of LV peak strain and clinical indices in T2DM with AR patients

	Radial PS				Circumferential PS				Longitudinal PS			
	Univariate	Multivariate			Univariate	Multivariate			Univariate	Multivariate		
	R	Β (R = 0.338)	P	95%CI	R	Β (R = 0.450)	P	95%CI	R	Β (R = 0.393)	P	95%CI
Regurgitation degree	− 0.579^*^	− 0.577	< 0.001	− 8.776 to − 5.627	− 0.653^*^	− 0.662	< 0.001	− 3.160 to − 4.487	− 0.608^*^	− 0.621	< 0.001	− 2.298 to -3.407
Gender^#^	− 0.059^*^	− 0.148	0.014	− 0.85 to − 7.543	− 0.055^*^	− 0.181	0.002	− 3.364 to − 0.814	− 0.022^*^	− 0.183	0.009	-2.766 to -0.408
Fasting plasma glucose	0.185^*^	0.248	0.169	–	0.125	0.1	0.652	–	− 0.183^*^	− 0.064	0.231	–
HbA1c %	− 0.122^*^	− 0.046	0.542	–	− 0.130	–	–	–	− 0.226^*^	− 0.195	0.187	–
Age (years)	− 0.19^*^	0.105	0.868	–	− 0.179^*^	0.042	0.811	–	0.178^*^	0.034	0.38	–
Years with diabetes	− 0.063	–	–	–	− 0.036	–	–	–	0.099	0.05	0.499	–
BMI (kg/m^2^)	− 0.032	–	–		− 0.016	–	–	–	0.123	0.098	0.142	–
SBP (mmHg)	0.071	–	–		− 0.050	–	–	–	0.061	–	–	–
DBP (mmHg)	− 0.093	–	–		− 0.074	–	–	–	0.141	–	–	–
TG (mmol/l)	− 0.003	–	–		− 0.006	–	–	–	− 0.003	–	–	–
TC (mmol/l)	0.143	–	–		0.192^*^	0.31	0.774	–	− 0.185^*^	− 0.105	0.130	–
HDL (mmol/l)	0.070	–	–		0.157^*^	1.001	0.078	–	− 0.033	–	–	–
LDL (mmol/l)	0.063	–	–		0.100	–	–	–	− 0.082	–	–	–

*P < 0.1 were included in the stepwise multiple linear regression model. ^#^For gender, we used 0 to represent for female and 1 to represent for male

*PS* peak strain, *HbA1c* glycated haemoglobin, *TC* total cholesterol, *TG* triglycerides, *HDL* high-density lipoprotein, *LDL* low-density lipoprotein, *CI* confidence intervals

After adjusting for age, sex, BMI, systolic blood pressure, and resting heart rate, the multiple linear regression analysis further showed that the regurgitation degree was an independent factor of LV global radial (β =  − 0.577), circumferential (β = − 0.662), and longitudinal (β = − 0.621) PS (all P < 0.001). Sex was found to be an independent factor of LV global radial (β = − 0.158), circumferential (β = − 0.181), and longitudinal (β = − 0.163) PS in patients with T2DM who presented with AR (Table 4).

### Reproducibility of LV PS

The intragroup correlation coefficient analysis revealed that the CMR tissue-tracking technique for measuring LV strain parameters had good intra- and interobserver consistency (Additional file 3: Table S1).

## Discussion

This study investigated the combined effects of AR on the CMR-derived LV structure and strain in diabetes. First, patients with T2DM had a lower PS (radial and longitudinal) and PDSR (radial, circumferential, and longitudinal) even without AR. Second, patients with T2DM who presented with AR had a significantly lower LV strain parameters in the three directions, and there was a decrease in LVEF and increase in LV mass. Third, along with the aggravation of regurgitation, the LV strain of patients with T2DM who presented with AR decreased progressively. Meanwhile, early diastolic dysfunction was observed in the mild AR group and further impairment in systolic function in the moderate and severe groups. Fourth, AR degree was independently correlated with LV PS (radial, circumferential, and longitudinal).

### Mechanical damage in the left ventricular myocardium caused by T2DM

Previous studies have shown the importance of CMR-derived global longitudinal PS in patients with T2DM. Habek et al. and Vukomanovic et al. revealed that strain in the longitudinal direction and in the diastolic period is initially impaired at the early stage [9, 20–22]. Moreover, early diastolic dysfunction and late systolic dysfunction will occur. The results of our previous study about myocardial changes in patients with T2DM without AR are consistent with those of the current research. This study found that patients without AR had a lower PS (radial and longitudinal) and PDSR in the three directions and a higher PSSR (radial and longitudinal) than healthy controls. However, they had similar LVEDV, LVESV, LVSV, and LVEF. Diabetes mellitus involves metabolic pathways, including alteration in fatty acid metabolism and chronic hyperglycemia-induced changes in circulating hormones and cytokines leading to impaired myocardial relaxation at the early stage. Early mild diastolic dysfunction can be identified; subsequently, systolic dysfunction will occur along with an increase in LV wall thickness and mass [11, 23]. Hence, potential early changes in the heart, including global and regional myocardial strains in the diastolic and systolic periods, can be accurately identified at the early stages of T2DM via CMR tissue tracking, even under conditions that include a preserved LVEF [24–26]. Another point is that patients without T2DM had a lower LV mass index than control. The reasons may be related to weight loss, medicine taking, and low-energy diet in diabetic patients [27–29].

### Additive effect of AR on left ventricular injury in patients with T2DM

Patients with T2DM (AR +) had a higher LV mass, LVEDV, and LVESV and a significantly lower LVEF than healthy controls and patients with T2DM (AR −). Moreover, the T2DM (AR +) group had a remarkedly lower LV global PS, PSSR, and PDSR in the three directions. Therefore, AR has an additive effect on LV deformation in patients with T2DM, particularly LV function impairment. A series of studies about nondiabetic AR have shown that global longitudinal strain decreases early in the course of the disease and is a marker of AR severity [19, 30, 31]. The pathologic model of AR is an increase in preload and afterload, leading to LV eccentric hypertrophy, progressive dilation, and systolic dysfunction, which manifest microscopically as cardiomyocyte apoptosis and myocardial interstitial fibrosis similar to the pathological process of simple diabetic cardiomyopathy [10, 32, 33]. The myocardium may present with a high LV volume and low LV compliance and may induce significant LV dysfunction under the double blow of diabetes and AR [3].

Based on the systolic and diastolic data, compared with the healthy control group, the T2DM (AR −) group experienced a significant decrease in PDSR in the three directions, and PSSR (radial and longitudinal) but not PSSR (circumferential). Patients with T2DM without AR first developed LV diastolic dysfunction due to subendocardial fiber damage. However, their systolic function remained within normal range at the early stages. Combined with AR, the systolic function of the left ventricle will significantly reduce. Then, LV dysfunction and heart failure may eventually develop. Therefore, AR can not only further aggravate LV diastolic dysfunction but also cause severe LV systolic dysfunction. Therefore, the development of AR must also be considered in the management of patients with diabetes due to these negative superimposed effects [3, 5, 34].

### Characteristics of LV strain injury caused by different regurgitation degrees

The different regurgitation degrees of patients with T2DM were compared. The results showed that the mild, moderate, and severe AR groups experienced a progressive decrease in LV global PS. The diastolic function of the mild AR group was impaired. Meanwhile, the systolic function of the moderate and severe groups decreased. A previous study revealed that damage in the LV caused by AR starts from diastolic function. With the aggravation of regurgitation, there was evident systolic dysfunction [35–37]. Therefore, mild AR in patients with diabetes should be emphasized to prevent irreparable damage to LV function. In addition, multivariate analysis revealed that AR degree was significantly associated with LV global radial, circumferential, and longitudinal PS in patients with diabetes. Further, PS might decrease in patients with concurrent diabetes and AR [5].

### Limitations

The current study had several limitations. First, although the sample size was large enough to confirm the additive effect of AR on LV impairment in patients with T2DM, this was a single-center study. Second, long-term follow-up data were not available, and there were limitations due to the cross-sectional nature of the study. Third, only patients with diabetes who presented with AR were assessed, and individuals with AR alone were not included. However, previous studies have assessed the effects of AR alone. Nevertheless, a comparative study including patients with AR alone should be performed.

## Conclusions

AR may aggravate LV stiffness and cause decreased LV strain and cardiac dysfunction in patients with T2DM. Regurgitation and sex were independently correlated with LV PS in patients with T2DM who presented with AR. The evaluation of LV strain may help clinicians monitor the progression of myocardial stiffness and use additional strategies to delay LV dysfunction in patients with T2DM who present with AR.

## Supplementary Information

**Additional file 1: Figure S1.** Analysis of left ventricular volume and function by cardiac magnetic resonance cine images. The left ventricular endocardium (red) and epicardium (green) were outlined on the left ventricular short axis images of end diastolic (A) and end systolic (B) according to the reference line (C), two-chamber long axis (D) and four-chamber long axis (E) images of end diastolic. The blue T-line defines the mitral plane and apex. (F) shows the 3D volume tissue tracking model of left ventricle automatically established.

**Additional file 2: Figure S2.** Ratio of the aortic jet width (diameter) to left ventricular outflow tract (LVOT) diameter. The diameter of the color jet is measured immediately beneath the aortic valve is a semi-quantitative index of aortic regurgitation severity. Parasternal short axis view (A); Parasternal long axis view (B).

**Additional file 3: Table S1.** Reproducibility about intragroup correlation coefficient of LV strains via CMR tissue-tracking technique.

## Data Availability

The datasets used and/or analyzed during the current study are available from the corresponding author on reasonable request.

## Abbreviations

AR: Aortic regurgitation
T2DM: Type 2 diabetes diabetes mellitus
LV: Left ventricle
CMR: Cardiac magnetic resonance
LVEF: Left ventricle ejection fraction
PS: Peak strain
HbA1c: Glycated haemoglobin
HDL: High density lipoprotein
LDL: Low density lipoprotein
BMI: Body mass index
RV: Right ventricle
EDV: End‑diastolic volume
ESV: End systolic volume
SV: Stroke volume
LVEF: Left ventricle ejection fraction
RF: Regurgitation fraction
PSSR: Peak systolic strain rate
PDSR: Peak diastolic strain rate
ICC: Intraclass correlation coefficient
