# Supplementary figures and images for: Additive effect of aortic regurgitation degree on left ventricular strain in patients with type 2 diabetes mellitus evaluated via cardiac magnetic resonance tissue tracking
Source: Cardiovasc Diabetol. 2022 Mar 11;21:37. doi: 10.1186/s12933-022-01471-2 (PMC8917654; doi:10.1186/s12933-022-01471-2)

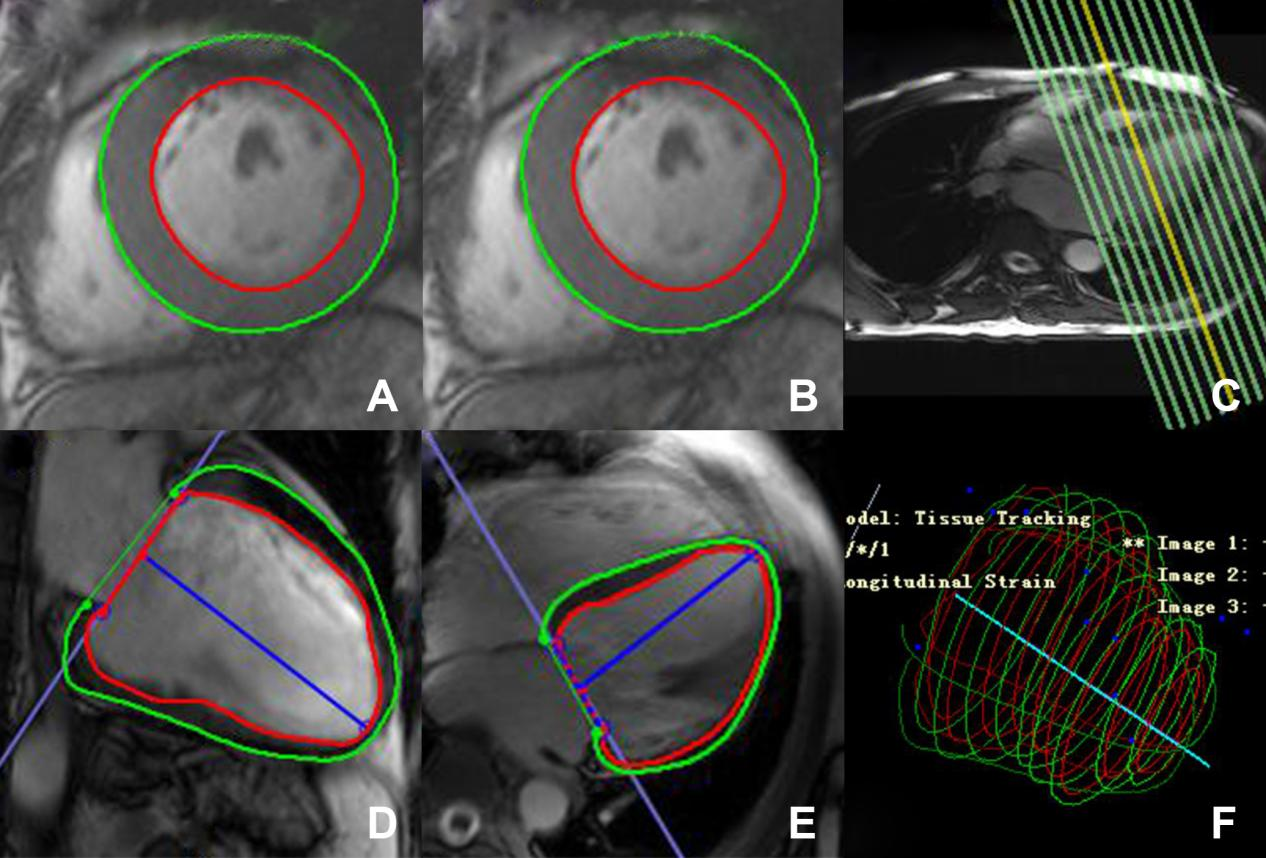

Supplement: Supplementary file 1 — Additional file 1: Figure S1. Analysis of left ventricular volume and function by cardiac magnetic resonance cine images. The left ventricular endocardium (red) and epicardium (green) were outlined on the left ventricular short axis images of end diastolic (A) and end systolic (B) according to the reference line (C), two-chamber long axis (D) and four-chamber long axis (E) images of end diastolic. The blue T-line defines the mitral plane and apex. (F) shows the 3D volume tissue tracking model of left ventricle automatically established. [file 12933_2022_1471_MOESM1_ESM.png]

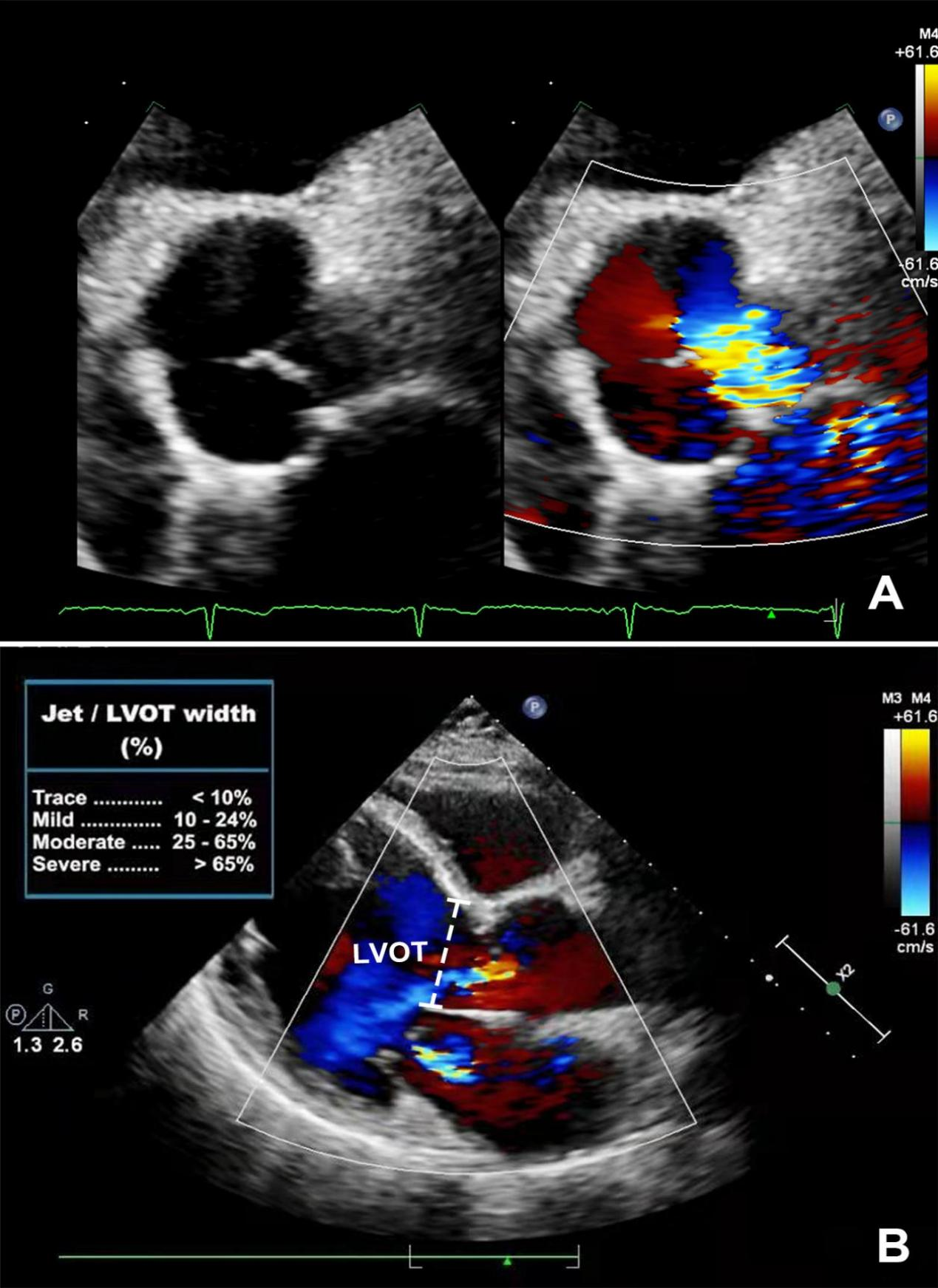

Supplement: Supplementary file 2 — Additional file 2: Figure S2. Ratio of the aortic jet width (diameter) to left ventricular outflow tract (LVOT) diameter. The diameter of the color jet is measured immediately beneath the aortic valve is a semi-quantitative index of aortic regurgitation severity. Parasternal short axis view (A); Parasternal long axis view (B). [file 12933_2022_1471_MOESM2_ESM.png]
