# Supplementary material for: Additive effect of aortic regurgitation degree on left ventricular strain in patients with type 2 diabetes mellitus evaluated via cardiac magnetic resonance tissue tracking
Source: Cardiovasc Diabetol. 2022 Mar 11;21:37. doi: 10.1186/s12933-022-01471-2 (PMC8917654; doi:10.1186/s12933-022-01471-2)
Supplement: Supplementary file 3 — Additional file 3: Table S1. Reproducibility about intragroup correlation coefficient of LV strains via CMR tissue-tracking technique. [file 12933_2022_1471_MOESM3_ESM.docx]

**Table S1. Reproducibility about intragroup correlation coefficient of LV strains via CMR tissue-tracking technique**

|  | intraobserver |  | interobserver |  |
| --- | --- | --- | --- | --- |
|  | ICC | 95%CI | ICC | 95%CI |
| Peak strain(%) |  |  |  |  |
| Radial | 0.943 | 0.916-0.965 | 0.849 | 0.814-0.901 |
| Circumferential | 0.921 | 0.902-0.949 | 0.793 | 0.754-0.831 |
| Longitudinal | 0.902 | 0.932-0.892 | 0.810 | 0.833-0.782 |
| PSSR(1/s) |  |  |  |  |
| Radial | 0.869 | 0.816-0.923 | 0.756 | 0.638-0.847 |
| Circumferential | 0.912 | 0.902-0.943 | 0.863 | 0.791-0.941 |
| Longitudinal | 0.962 | 0.957-0.969 | 0.935 | 0.918-0.951 |
| PDSR(1/s) |  |  |  |  |
| Radial | 0.873 | 0.824-0.923 | 0.727 | 0.620-0.834 |
| Circumferential | 0.866 | 0.821-0.925 | 0.719 | 0.614-0.823 |
| Longitudinal | 0.928 | 0.921–0.952 | 0.879 | 0.825–0.913 |

ICC intragroup correlation coefficient; CI confidence interval; PSSR peak systolic strain rate; PDSR peak diastolic strain rate
